# Micro-rocket robot with all-optic actuating and tracking in blood

**DOI:** 10.1038/s41377-020-0323-y

**Published:** 2020-05-11

**Authors:** Dengfeng Li, Chao Liu, Yuanyuan Yang, Lidai Wang, Yajing Shen

**Affiliations:** 1 Department of Biomedical Engineering, City University of Hong Kong, 999077 Hong Kong, China; 2 City University of Hong Kong Shenzhen Research Institute, Shenzhen, 518057 China

**Keywords:** Polymers, Imaging and sensing

## Abstract

Micro/nanorobots have long been expected to reach all parts of the human body through blood vessels for medical treatment or surgery. However, in the current stage, it is still challenging to drive a microrobot in viscous media at high speed and difficult to observe the shape and position of a single microrobot once it enters the bloodstream. Here, we propose a new micro-rocket robot and an all-optic driving and imaging system that can actuate and track it in blood with microscale resolution. To achieve a high driving force, we engineer the microrobot to have a rocket-like triple-tube structure. Owing to the interface design, the 3D-printed micro-rocket can reach a moving speed of 2.8 mm/s (62 body lengths per second) under near-infrared light actuation in a blood-mimicking viscous glycerol solution. We also show that the micro-rocket robot is successfully tracked at a 3.2-µm resolution with an optical-resolution photoacoustic microscope in blood. This work paves the way for microrobot design, actuation, and tracking in the blood environment, which may broaden the scope of microrobotic applications in the biomedical field.

## Biotechnology: Laser-powered robots rocket through the bloodstream

Researchers now report that lasers can be used to safely move tiny robots in the blood. Dr. Dengfeng Li and Mr. Chao Liu from the City University of Hong Kong in China and colleagues used 3D printing to create microscale tubes, connected to resemble a central rocket with two side boosters attached. After applying a light-absorbing gold coating to the micro-rockets, the team irradiated the device with laser pulses. The resulting thermal gradient along the length of the tubes propelled the device steadily through solutions with high viscosity, such as blood. Furthermore, the gold coating generates ultrasonic signals that can be spotted through photoacoustic microscopic techniques. Injections of the micro-rockets into blood revealed they could be tracked with microscale resolution as they were directed to flow through the bloodstream.

## Introduction

The development of autonomous artificial micro/nanorobots has attracted considerable attention due to their potential in various biomedical applications, such as in vivo treatment or surgery^1^. In recent years, scientists have desired that microrobots be able to reach all parts of the human body through blood vessels to assess or treat diseases in different organs^2^. In the current stage, drug delivery and tumor treatment relying on microrobots are mostly carried out in the stomach, intestinal tract, or subcutaneous tissue^3–6^. To assist robots in entering other organs, blood vessels are the best channel, as blood is circulated throughout the body. For the time being, however, the development of microrobots that can work in the blood faces many challenges, including the achievement of effective actuation and precise observation, which become more serious for a robot with a size of less than 100 µm.

Different from the open environment in a lab, a microrobot experiences harsh situations in blood, which is a viscous and fast-flowing environment that can be difficult to swim through. Although chemical-driven microrobots are capable of high-speed motion, the required chemical fuels are often toxic; hence, they cannot be used in blood vessels^7^. Magnetic field actuation often shows good biological compatibility due to its noninvasive peculiarity and good controllability in viscous liquids^8,9^. However, the corresponding low peak power and moving speed prevent its application in blood vessels, even in the slowest capillaries^10^. Among the nontoxic noninvasive physical field actuations^11,12^, light-driven microrobots often tend to exhibit high-speed motion, and researchers have proved the powerful capability of light-driven microrobots to penetrate the cell membrane under pulsed light excitation^13,14^. The flexibility of the modulation of the wavelength, frequency, and intensity of light will increase the applicability of light-driven microrobots. Moreover, effective light actuation requires only asymmetry in the robotic structure or actuation area, offering more flexibility for the structural design of a robot.

In addition to actuation, another challenge is the precise tracking of a microrobot in the bloodstream^15^. To date, many imaging techniques, such as X-ray imaging, CT imaging, MR imaging, fluorescence imaging, and ultrasound imaging, have been tested for microrobot observation in living tissue^16^. Even though these methods can find the swarm location of microrobots in living animals, it is still difficult to image and track a single microrobot in vivo with high resolution. As a new imaging method, photoacoustic (PA) tomography has the advantages of high resolution and optical-absorption contrast, and has demonstrated its potential for tracking microcapsules full of spherical microrobots in the mouse intestine or micro objects in ex vivo tissues^17–19^. However, due to the poor optical absorption of microrobots and unoptimized imaging sensitivity, photoacoustic imaging of a single microrobot has not been implemented in blood. To precisely track the motion of a microrobot in a blood vessel, it is essential to engineer the microrobot and photoacoustic imaging system such that the imaging sensitivity is improved to a micrometer resolution^20^.

According to nature, the interface plays a crucial role in reinforcing the interaction efficiency. For instance, lotuses realize strong buoyancy on the surface of water by large numbers of micro- and nanoscopic architectures on their leaf surfaces, which increase the contact area and greatly increase the interaction efficiency between the organism and matter^21^. The optimized contact interface is expected to be very beneficial in building up the propulsion efficiency of microrobots. Herein, inspired by the interface enhancement concept, we design a three-dimensional (3D) structure with multinozzles on a robot to speed up the light-driven efficiency. The multi-nozzle structure increases the quantity of the propulsion channel and the light-excitation area and leads to a threefold speed increase. In addition to the effective motion in a viscous fluid, this micro-rocket can also be tracked by optical-resolution photoacoustic imaging in blood with a 3.2-µm resolution.

## Results

### System of photoacoustic imaging for single-microrobot tracking

**Fig. 1:**
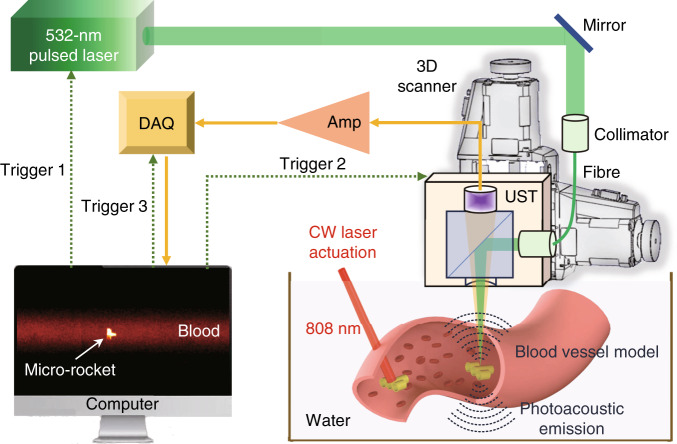
Schematic illustration of the micro-rocket robot with all-optic actuating and tracking carried out in the blood. The micro-rocket is actuated by a near-infrared continuous wave (CW) laser to realize forward motion in the blood vessel. An 8-kHz 50-nJ pulse laser with a wavelength of 532 nm is used for photoacoustic imaging. The rocket’s attitude and position can be observed with a lateral resolution of 3.2 µm based on an optical-resolution photoacoustic system. Amp amplifier, DAQ data acquisition, UST ultrasound transducer

We develop an optical-resolution photoacoustic microscopy (OR-PAM) system to track a single microrobot in the bloodstream (Fig. 1). When a short-pulsed laser beam irradiates light-absorbing materials, the absorbed energy will be converted into ultrasonic emission. In the OR-PAM system, a nanosecond pulsed laser beam (532 nm) is coupled into a 2-m single-mode fiber as the light source. After the fiber, the laser beam is focused by an objective lens, is reflected with a prism, and illuminates the sample to generate the photoacoustic wave. The photoacoustic wave is collected by a concave acoustic lens, and detected by a piezoelectric ultrasound transducer. Volumetric images are obtained by raster scanning of the OR-PAM probe. The lateral resolution of this OR-PAM system is 3.2 µm (Supplementary Fig. S1). With a layer of gold coating, the micro-rocket can generate stronger photoacoustic signals than the blood at 532 nm, which guarantees the imaging contrast of the micro-rocket in the blood. Under the illumination of a quasi-CW 808-nm laser, the micro-rocket can realize fast motion in a rubber vessel simulating the vascular scene. At the same time, the OR-PAM system provides high-resolution tracking of the micro-rocket.

### Micro-rocket with multinozzles

**Fig. 2:**
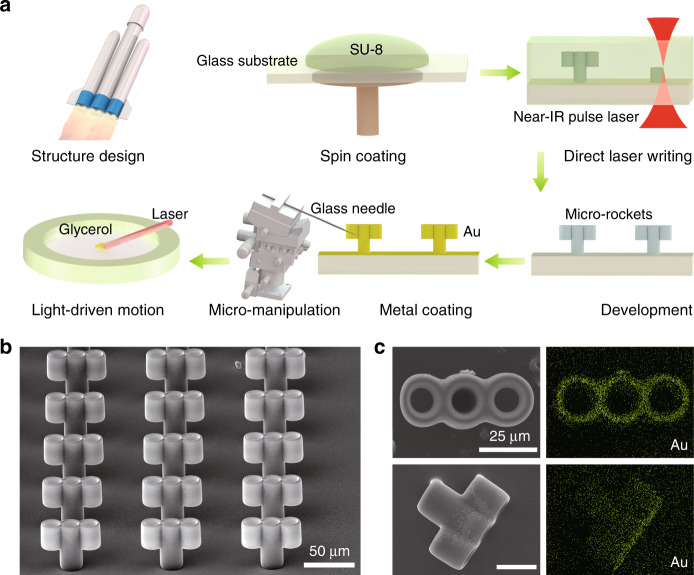
Design and fabrication of interface-enhanced micro-rockets by 3D printing. **a** Schematic of the 3D microprinting process. Direct laser writing for 3D microprinting with a nanoscale resolution; the 3D structures are acquired after developing the uncross-linked SU-8 photoresist; a gold layer with a thickness of 100 nm is coated onto the micro-rocket; and the micro-rockets are transferred via micro-manipulation to the liquid solution for further light-driven motion. **b** SEM images of the micro-rocket array. Inspired by the interface enhancement in nature, the micro-rocket is designed with three tubes with a diameter of 20 µm and a wall thickness of 2 µm. The lengths of the central tube and two symmetrical tubes are 45 µm and 22.5 µm, respectively. **c** The top view and side view of the energy dispersive X-ray spectrum (EDS) mapping of the micro-rockets

To effectively move in highly viscous blood, the robot needs to generate a high instantaneous propelling force. Inspired by the interface enhancement, a rocket-shaped structure with three nozzles at one end is designed. The microscale rocket is fabricated by 3D printing (Fig. 2a). This direct laser writing technique can achieve nanoscale resolution, making it suitable for fabricating 3D hollow microstructures. On a transparent glass substrate, SU-8 is printed with 400-nm slicing and 400-nm filling (Supplementary Fig. S2), and the cross-linked structures stand as an array (Fig. 2b) after 3D printing and development. The microstructure is composed of three tubes with a diameter of 20 µm and a wall thickness of 2 µm. The lengths of the central tube and two symmetrical tubes are 45 µm and 22.5 µm, respectively (Supplementary Fig. S3).

To enhance the driving force and photoacoustic imaging sensitivity, a 100-nm-thick gold layer is coated onto the microrobots. Figure 2c demonstrates the uniform gold coating via energy dispersive X-ray spectrum (EDS) mapping as viewed from the top and side of the micro-rocket. The functional layer on the surface of the micro-rocket, i.e., the gold layer, exhibits strong absorption in the visible and near-infrared bands (Supplementary Fig. S6), and thus generates strong thermal energy that drives the micro-rocket forward upon quasi-CW 808-nm irradiation. In imaging, it also generates strong photoacoustic signals upon nanosecond 532-nm optical excitation.

### Light actuation mechanism of the microrobot with multiple nozzles

**Fig. 3:**
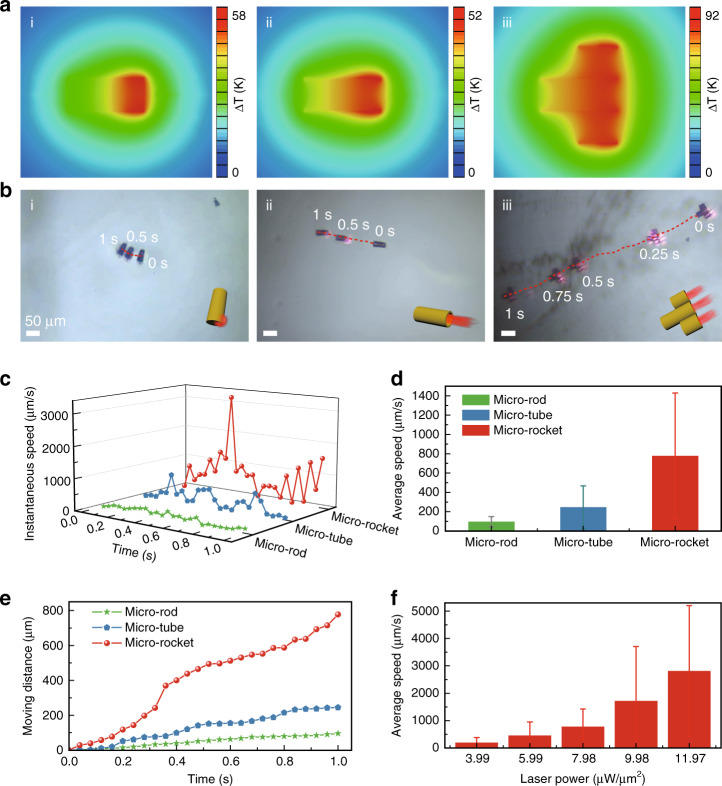
Ultrafast motion of the light-driven micro-rocket in a viscous liquid. **a** Theoretical simulation of the microrobots under near-infrared light excitation. The steady distribution of temperature on the micro-rod robot (i), micro-tube robot (ii) and micro-rocket robot (iii). **b** Movement routes of the micro-rod, micro-tube, and micro-rocket robots in a 50% glycerol solution under near-infrared light actuation. Scale bar: 50 µm. **c** Instantaneous moving speed of the three microrobots within 1 s. **d** Average speed of the three microrobots. The micro-tube robot moves at an average speed of 244.6 µm/s, 2.5 times faster than the micro-rod robot, due to the actuation of heat flow from the tube structure. The average speed of the micro-rocket robot reaches 777.4 µm/s, approximately three times that of the micro-tube robot, benefiting from the multiple tubular channels. **e** Moving distance of the three microrobots. **f** Average speed of the micro-rocket robot under different power laser actuations (2.8 mm/s under 11.97 µW/µm^2^)

The micro-rocket is actuated based on the photothermal mechanism. Under the excitation of a 1-W 808-nm laser with an ~354-µm focus size (Supplementary Fig. S8), photothermal heat is generated from the Au layer on the microrobots. When the near-infrared laser excites the tail of the micro-rod robot, finite element analysis (FEA) shows that the tail’s temperature is significantly higher than that of the head. The thermophoretic force from the asymmetric temperature gradient directly pushes the micro-rod robot forward (Fig. 3a, i)^22,23^. For the micro-tube and micro-rocket robot, in addition to the self-thermophoresis force, a strong heat flow exits the tubular structure due to the photothermal energy concentration inside the tube (Fig. 3a, ii and iii)^14^. Based on the simulation results, the micro-tube robot is expected to run faster than the micro-rod robot due to the introduction of the tubular structure, and the micro-rocket should have the fastest moving speed among them due to the advantages of the multiple channels. Moreover, as the light power increases, the temperature of the micro-rocket body will also increase, resulting in a faster motion speed (Supplementary Fig. S5).

For comparison, the micro-tube and micro-rod robots were fabricated to verify the advantages of the tubular robot and multichannel robot (Supplementary Figs. S3 and S4). To achieve controllable light-driven motion, the microrobots were transferred from the substrate into an acrylic container full of 50% glycerol solution via a glass micro-needle based on a micro-manipulation system (Supplementary Fig. S7)^24^. The experimental results indicate that in 50% glycerol solution under 1-W 808-nm laser irradiation with a power density of 7.98 µW/µm^2^, the micro-rocket travels 777.4 µm within 1 s, being approximately threefold faster than the micro-tube robot and 7.5 times faster than the micro-rod robot, benefiting from the multiple tubular channels (Fig. 3d, e; Supplementary Movie S1). These results agree well with the theoretical prediction.

### High-speed motion in a highly viscous solution

The viscous liquid, i.e., 50% glycerol solution, is used to imitate the high-viscosity environment in the human body. The viscosity of the 50% glycerol solution is 4.21 mPa‧s, which is very close to that of human blood (3–4 mPa‧s). In this viscous liquid, it is impressive that the instantaneous moving speed of the micro-rocket exceeds 3 mm/s under 1-W 808-nm laser actuation (Fig. 3c), being much faster than the micro-rod and micro-tube robots owing to the enhanced interface design with multiple nozzles. This energetic instantaneous power allows the micro-rocket to break through the viscous force of the viscous fluid. Moreover, the micro-rocket presents a higher moving speed as the actuated laser power increases (Fig. 3f; Supplementary Movie S2). Specifically, under the excitation of the 1.5-W laser, the average moving speed reaches a fairly impressive 2.8 mm/s (62 body lengths per second), which serves as the baseline speed for rocket usage in the human body. Compared with the microrobots actuated by other methods, our design exhibits obvious advances in the motion speed (Supplementary Table S1), especially in instant power generation, offering the basis for the possible effective motion in real body fluids.

**Fig. 4:**
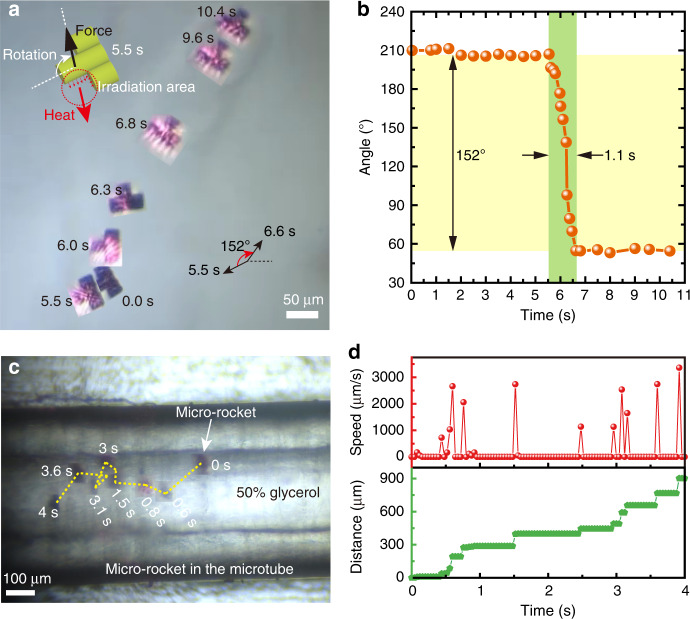
Motion control of the rocket robot in free space and fast actuation in a blood-vessel-like microtube. **a** The turning motion of the micro-rocket in 50% glycerol in free space controlled by near-infrared light. The light excites the side face of the micro-rocket to achieve the turning movement. **b** The relationship between the micro-rocket moving angle and the moving time. Turning of more than 150° is realized within 1.1 s. **c** Fast motion of the micro-rocket in the microtube. A microtube filled with a 50% glycerol solution was used to simulate a blood vessel. **d** Instantaneous moving speed and moving distance of the micro-rocket in the microtube

The swimming direction of the micro-rocket is adjusted by the laser excitation position in a controllable manner. When the laser irradiates the whole tail of the micro-rocket, the rocket moves in a straight line (Fig. 3b). When the laser beam irradiates only one side of the rocket’s head or tail, the asymmetric heat flow between the two sides will cause rotation of the rocket body (Fig. 4a). Then, the micro-rocket can run along a straight line again by redirecting the irradiation area of the laser to once again cover the rocket’s tail. Figure 4a, b shows that the micro-rocket realizes 152° turning within 1.1 s (Supplementary Movie S3). This controlled movement, to a large extent, improves the applicability of the micro-rocket, which provides the possibility of targeted biomedical treatment.

To simulate the micro-rocket’s motion in a static blood vessel, a rubber microtube with an inner diameter of 250 µm full of 50% glycerol is used as the container (Fig. 4c). The micro-rockets are first transferred to the entrance of an empty clean rubber tube by the micro-manipulated glass needle, and then a drop of dilute glycerol solution is dripped onto the tube opening to bring the micro-rockets into the rubber tube based on the capillary effect. Under the microscope, the controlled 808-nm beam easily reaches the micro-rocket through the transparent tube. In the rubber tube, the micro-rocket moves rapidly at an average moving speed of 225.3 µm/s and a maximum instantaneous speed of 3.4 mm/s (Fig. 4d; Supplementary Movie S4). As far as we know, this is the highest recorded speed of the ~50-µm microrobot in such a viscous liquid tube. The ability to generate high instantaneous power allows the micro-rocket to move freely in the viscous fluid.

The inner surface of the lumen of a human body vessel is generally highly lubricated. Only when the vessel is diseased, such as the case of arteriosclerosis, may the internal lubricity of blood vessels change. Therefore, what hinders the motion of the robot is the fast blood flow and high blood viscosity instead of the friction or adhesion from the vascular inner wall. More importantly, the distribution of the blood flow in blood vessels is parabolic. The closer to the middle of the vessel, the faster the blood flow is. It may be a good idea to constrain the microrobot near the surface of the vessel by an additional force, such as magnetic attraction, to move against the blood flow.

### Photoacoustic tracking of a single micro-rocket

**Fig. 5:**
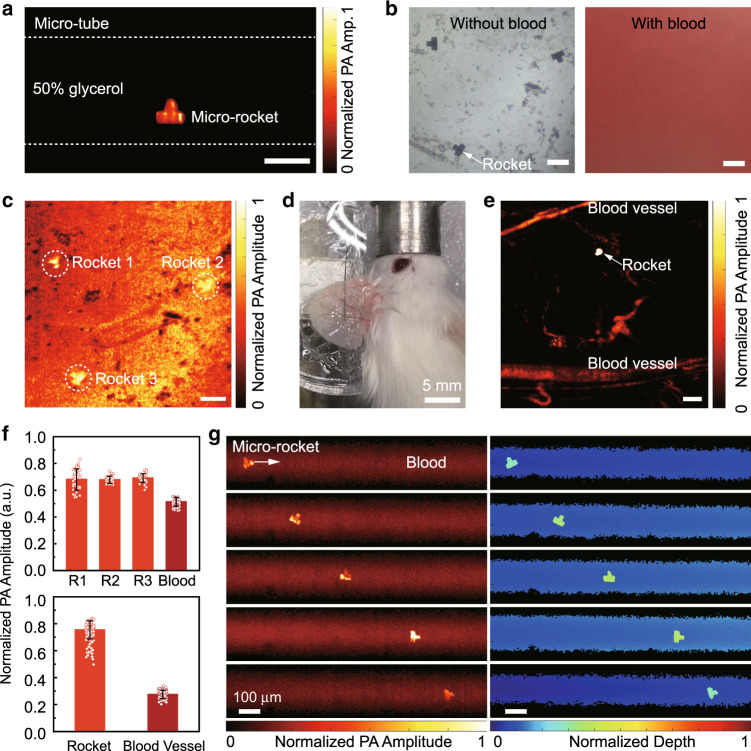
Photoacoustic imaging of a single micro-rocket in blood, and penetration of living mouse ear. **a** Clear high-resolution PA imaging of a single micro-rocket in the transparent solution. **b** Optical microscopy of the micro-rockets without blood and within blood. The optical microscope fails to observe the micro-rockets covered by 500-µm-thick bovine blood. **c** Corresponding visible PA images of these micro-rockets in thick blood. **d** Mouse ear covering a micro-rocket. **e** PA imaging of the micro-rocket through the living mouse ear. **f** Contrast of the normalized PA amplitude between the micro-rockets and the blood or the living blood vessel. **g** High-resolution PA tracking of a single micro-rocket in the blood vessel model, i.e., a 250-µm-diameter rubber tube filled with bovine blood. (Left) PA imaging with the intensity distribution. (Right) PA imaging with the depth distribution. Combining the structure and depth information, 3D tracking of a single light-driven micro-rocket is realized in the bloodstream. Scale bar: 100 µm for **a**, **b**, **c**, **e**, and **g**

We employed OR-PAM to image the micro-rocket in both a clear medium and blood. In a transparent rubber tube filled with glycerol solution, the 532-nm laser beam irradiates the micro-rocket to acquire PA images (Fig. 5a). Then, we cover the microrobot with 500-μm-thick bovine blood, with the micro-rocket being visible in the PA images (Fig. 5c). The average PA amplitude of the micro-rocket is 33% higher than that of the background blood (Fig. 5f), and thus, the rockets can be well distinguished from the blood. For comparison, we used a conventional optical microscope to image the microrobot in both a clear medium and blood. Although the microrobot can be resolved in a transparent solution, it becomes almost invisible when the micro-rocket is covered by blood (Fig. 5b). This shows the advantage of the PA imaging of microrobots in blood.

Considering that in the real environment of a living body, the blood vessels are covered by tissue, we placed the micro-rocket underneath the ear of an anaesthetized mouse (Fig. 5d). Figure 5e shows that high-contrast PA imaging of the micro-rocket can be achieved under the mouse ear. The normalized PA amplitude of the rocket covered by the mouse ear is three times higher than that of the background blood (Fig. 5f). Compared with the experiment in which the rocket is covered by blood, rocket imaging under the mouse ear yields a higher contrast. There are two possible reasons for this outcome. One is that in the blood-covering experiment, the blood above the rocket severely attenuates the incident light on the micro-rocket and thus lowers the contrast of the micro-rocket in PA images. The other reason is that in the ear-covering experiment, the PA probe is focused at the micro-rocket depth, while the blood vessels are off-focus, which reduces the PA signal of the blood vessels and thus increases the micro-rocket contrast in the PA images. This scenario indicates that the rocket can be detected in subcutaneous tissue.

To mimic the tracking of the micro-rocket in vessels, the micro-rocket was put into a 250-µm-diameter rubber tube filled with bovine blood. Using the OR-PAM system, we observed the ~50 µm micro-rocket in the blood tube (Fig. 5g). Both the structure and depth information of the single micro-rocket could be determined. When the micro-rocket moved in the rubber tube, the OR-PAM acquired multiple images at different positions (Supplementary Movie S5). Figure 5g shows the high-resolution PA images of the micro-rocket. During the movement, the depth of the rocket in the tube also changed. Therefore, combining the structure and depth information, OR-PAM can realize 3D tracking of a single light-driven micro-rocket in the bloodstream.

When the rocket is imaged in a blood vessel, the blood and the tissue will inevitably scatter the incident light, which will reduce the image contrast. In the future, a near-infrared laser can be used to increase the imaging contrast due to the lower absorption of the blood at near-infrared wavelengths. Meanwhile, because longer wavelengths have weaker scattering, near-infrared photoacoustic excitation can also increase the penetration depth. In addition, in the future, a fast OR-PAM system can be used for real-time tracking of the micro-rocket^25–28^.

## Discussion

Blood vessels are good channels through which microrobots can be transported to remote sites in the human body. The blood flow can provide part of the driving force for the microrobots in some cases. Of course, the microrobots should be capable of moving autonomously in a blood vessel under external actuation in addition to passively flowing with the bloodstream. In the future, microrobots may reach every part of the human body through blood flow circulation. To realize this difficult task, two basic challenges should be addressed: (i) high moving speed to overcome viscous fluid resistance and (ii) high-resolution imaging to observe a single microrobot in blood or tissue.

With high instantaneous driving energy, light actuation is an ideal choice for biomedical microrobot design. Here, a 3D-printed tubular micro-rocket is developed as a microrobot capable of moving quickly in a viscous fluid. The Au layer was used as the light-absorption layer for actuation and photoacoustic tracking. Generally, tubular microrobots often achieve a high moving speed in a liquid. Inspired by the important role of the surface interface in nature, we designed a microrobot with rocket-like multinozzles. Consequently, the moving speed of the micro-rocket could reach 2.8 mm/s in viscous 50% glycerol solution with a similar viscosity to that of human blood. Moreover, light excitation enabled the micro-rocket to break away from the rubber tube wall and realize effective motion in this blood vessel model. The high-performance motion of the light-driven micro-rocket in viscous fluid opens the door for its future biomedical applications in the human body.

With a high resolution of several micrometers, OR-PAM can observe a single micro-rocket in the bloodstream. To simulate the biological environment, a series of experiments under different imaging conditions were carried out. First, immersed in blood, the positions of several micro-rockets could be recognized by the significantly comparable photoacoustic signal between the micro-rockets and the blood. Then, through a living mouse ear, the micro-rocket still presented an obvious outline via the photoacoustic imaging method. Finally, a rubber tube full of bovine blood was used to model a blood vessel. Under near-infrared light actuation, the single micro-rocket could realize effective motion, and clear photoacoustic tracking of the robot could be achieved in the blood vessel model. As a result, optical-resolution photoacoustic imaging provides a new high-resolution biomedical imaging method for microrobot observation.

Although the developed OR-PAM can achieve high-resolution imaging of a microrobot in blood or underneath tissue, it is worth noting that the current system works only for shallow tissue, i.e., <~1 mm. In clinical-oriented applications, two methods might be used in the future to increase the penetration depth of OR-PAM. One is to use infrared light in the process of photoacoustic excitation. The other method is to use photoacoustic endoscopy to image microrobots in deep tissues^29–31^. Taking advantage of scalable resolutions and depths, photoacoustic imaging of the micro-rocket robot in deep tissue may also be possible in the future using a photoacoustic computed tomographic system^32^. In addition, please note that real-time tracking with high resolution remains difficult to achieve by the current system due to its slow imaging speed, which can be improved by developing faster pulsed lasers and OR-PAM scanners^26,27^. In addition, the intensity of the actuation laser exceeds the safety threshold for in vivo applications, which is an open problem for light-driven microrobots. Therefore, we use a structural design to accelerate the robot and make it able to achieve fast motion under weak driving light. As future work, more efforts should be carried out in regard to real-time optical-resolution photoacoustic microscopy and the fast light actuation of microrobots within the threshold range.

There are still some challenges to applying the proposed device in preclinical or clinical practice. First, biological tissue scatters light, which limits the depth of microrobotic driving and imaging. In the future, other techniques, such as mini-invasive laparoscopy or endoscopy, need to be developed to deliver light beyond superficial tissue^33^. Second, local thermal damage is inevitable due to photothermal driving. How to avoid or reduce local tissue damage needs to be studied in the future. For instance, delivering light using an optical fiber or an endoscopic device may reduce the optical pathlength in tissue and lower the possibility of tissue damage. Another possible approach that can be explored in the future is to cool the tissue before heating so that the thermal damage to the tissue can be mitigated^34^. Finally, although the micro-rocket can move through trunk blood vessels, it is too large to pass through arterioles and capillaries. To avoid clogging these vessels, two approaches can be explored in the future. One is to use biodegradable materials to fabricate the microrobots. When a robot reaches a small vessel, it can undergo a certain amount of degradation for a period of time. The other is to reduce the robot size such that it is smaller than the capillary diameter.

Although medical micro/nanorobots have achieved considerable advances, many aspects of research are still needed for safe and reliable micro/nanorobot-assisted clinical therapeutics in the human body. Future advances in materials science in terms of biocompatibility and biodegradability would intuitively improve the safety of the robot itself in the human body^35–37^. A sophisticated integrated system or platform for in vivo robot actuation, control, and medical imaging is also in high demand for real-world clinical-level applications. Importantly, given the extreme complexity of the human environment, determining how a robot can cross biological barriers to reach a diseased site will be a key challenge to overcome^37^.

In conclusion, we developed a 3D-printed ultrafast micro-rocket and realized effective motion and high-resolution photoacoustic tracking in viscous blood. Under the actuation of near-infrared light, the high-speed motion (2.8 mm/s) and powerful energy of the micro-rocket increase the possibility for microrobot applications in harsh environments in vivo. More importantly, OR-PAM successfully realizes the clear tracking and observation of a single micro-rocket in a blood vessel with a resolution of 3.2 µm. This work greatly improves the possibility of biomedical applications of microrobots in the human body.

## Materials and methods

### 3D printing of micro-rockets

The 3D micro-rocket was printed based on the direct laser writing technique by the Photonic Professional GT system (Nanoscribe GmbH). With a spin-coated 50-μm-thick photoresist (SU-8 50, MicroChem Corp.) on transparent glass and utilization of the pre-bake and soft-bake processes, a 780-nm laser was focused in the photoresist, and the structure was printed with 0.4-μm slicing and 0.4-μm filling. After the microprinting and post-bake processes, the sample was developed in an SU-8 developer (MicroChem Corp.) and rinsed with isopropyl alcohol. After air drying, hundreds of micro-rockets were obtained from the substrate. To realize near-infrared light actuation, a 100-nm-thick gold layer was coated on the micro-rocket surface.

### Characterization

The microstructures of the 3D-printed micro-rockets were observed by using a field scanning electron microscope (FSEM, FEI Quanta 450). The micro-rockets were transferred to a new clean substrate for the element distribution test. A scanning electron microscope (JEOL/JSM-5600) with an energy dispersive X-ray spectrum (OXFORD #INCA Energy 200 system-IE200C) was used for element mapping.

### FEA of light-driven micro-rocket

Finite element analysis (FEA) was used to analyze the temperature distribution of the micro-rocket under near-infrared light irradiation. The thermal conductivity, specific heat capacity, and density of the gold (Au) coating were 315 W/(m‧K), 0.13 kJ/(kg K), and 19300 kg/m^3^, respectively, and those for SU-8 were 0.2 W/(m‧K), 1.5 kJ/(kg K), and 1190 kg/m^3^. The laser with a power of 7.98 μW/μm^2^ was used to irradiate a quarter of the microrobot body in the tail region. The radiation time was set as 0.1 s.

### Light actuation

The 3D-printed micro-rockets were transferred to an acrylic-based container or a microtube full of glycerol solution by the micro-manipulation system. Glycerol (50%) was used as the viscous fluid medium to imitate the high-viscosity environment in the human body. A near-infrared laser with a wavelength of 808 nm was used as the light source to actuate the micro-rocket.

### Optical-resolution photoacoustic microscopy

A pulsed laser (VPFL-G-20, Spectra-Physics; operating wavelength: 532 nm; pulse duration: 7 ns; pulse-repetition rate: 1 MHz) was used as the light source. The laser was sent to an ultrasonically and optically confocal OR-PAM probe through a 2-m single-mode fiber (P1-460B-FC-2, Thorlabs Inc). In the OR-PAM probe, the beam was focused onto the biological tissue to generate the photoacoustic signals. In the experiments, a water tank full of deionized water was placed on top of the samples to carry out the lossless propagation of the ultrasonic waves considering that the impendence of water is similar to that of body tissue. A 50-MHz (V214-BC-RM, Olympus) ultrasound transducer inside the probe was placed ultrasonically and optically confocal above the laser beam to acquire the optimized PA signal. Detailed information on the OR-PAM probe can be found in previous papers^25,38–40^. A 2D photoacoustic image could be acquired after combining with a motorized two-dimensional scanner.

## Supplementary information

Supplementary Information for Micro-rocket robot with all-optic actuating and tracking in blood

Movie S1

Movie S2

Movie S3

Movie S4

Movie S5
