# Supplementary material for: Micro-rocket robot with all-optic actuating and tracking in blood
Source: Light Sci Appl. 2020 May 11;9:84. doi: 10.1038/s41377-020-0323-y (PMC7214411; doi:10.1038/s41377-020-0323-y)
Supplement: Supplementary file 1 — Supplementary Information for Micro-rocket robot with all-optic actuating and tracking in blood [file 41377_2020_323_MOESM1_ESM.docx]

Supplementary Information for

**Micro-rocket robot with all-optic actuating and tracking in blood**

Dengfeng Li^1^, Chao Liu^1^, Yuanyuan Yang^1^, Lidai Wang^1,2*^, Yajing Shen^1,2*^

1 Department of Biomedical Engineering, City University of Hong Kong, 999077 Hong Kong SAR, China.

2 City University of Hong Kong Shenzhen Research Institute, Shenzhen 518057, China.

These authors contributed equally: Dengfeng Li, Chao Liu

*Correspondence: lidawang@cityu.edu.hk (L.W.); yajishen@cityu.edu.hk (Y.S.)

**Supplementary Movies:**

**Movie S1.** Motion of the near-infrared light-driven micro-rod, micro-tube and micro-rocket.

**Movie S2.** Movement of the micro-rocket under different laser power actuation.

**Movie S3.** Controllable motion of the light-driven micro-rocket.

**Movie S4.** Fast motion in the microtube full of viscous 50% glycerol solution.

**Movie S5.** A single micro-rocket with optical-resolution photoacoustic tracking in blood.

**Tab. S1.** Summary of fast microrobots actuated by different fields.

| **Actuation field** | **Body length (µm)** | **Moving speed (µm‧s^-1^)** | **Medium** |
| --- | --- | --- | --- |
| Magnetic field | 35~60 | 250~320 | Water ^1,2^ |
| Chemical field | 12~50 | 275~2500 | 5%~15% H_2_O_2_ ^3,4^ |
| Ultrasound field | 2~180 | 200~1200 | PBS buffer solution, PEG ^5,6^ |
| Light field | 10~12 | 20~160 | Water ^7,8^ |
| This work | 45 | 2810 | 50% glycerol |


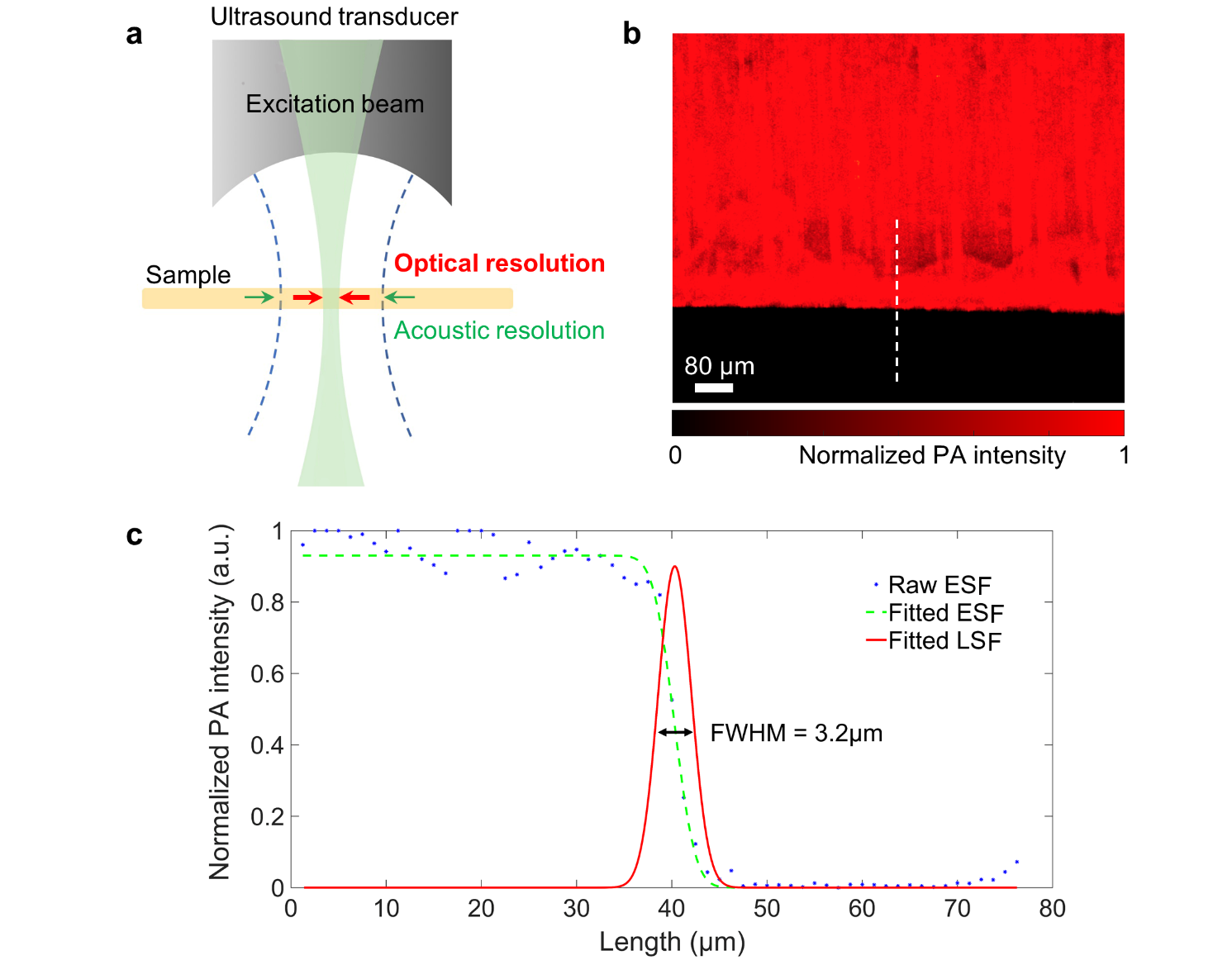


**Fig. S1**. Resolution test of the optical-resolution photoacoustic microscopy system. (a) Illustration of optical resolution and acoustic resolution. (b) Photoacoustic image of a sharp blade. (c) The line spread function (LSF) is calculated from the photoacoustic amplitude profile normal to a sharp blade edge. Lateral resolution is 3.2 µm.


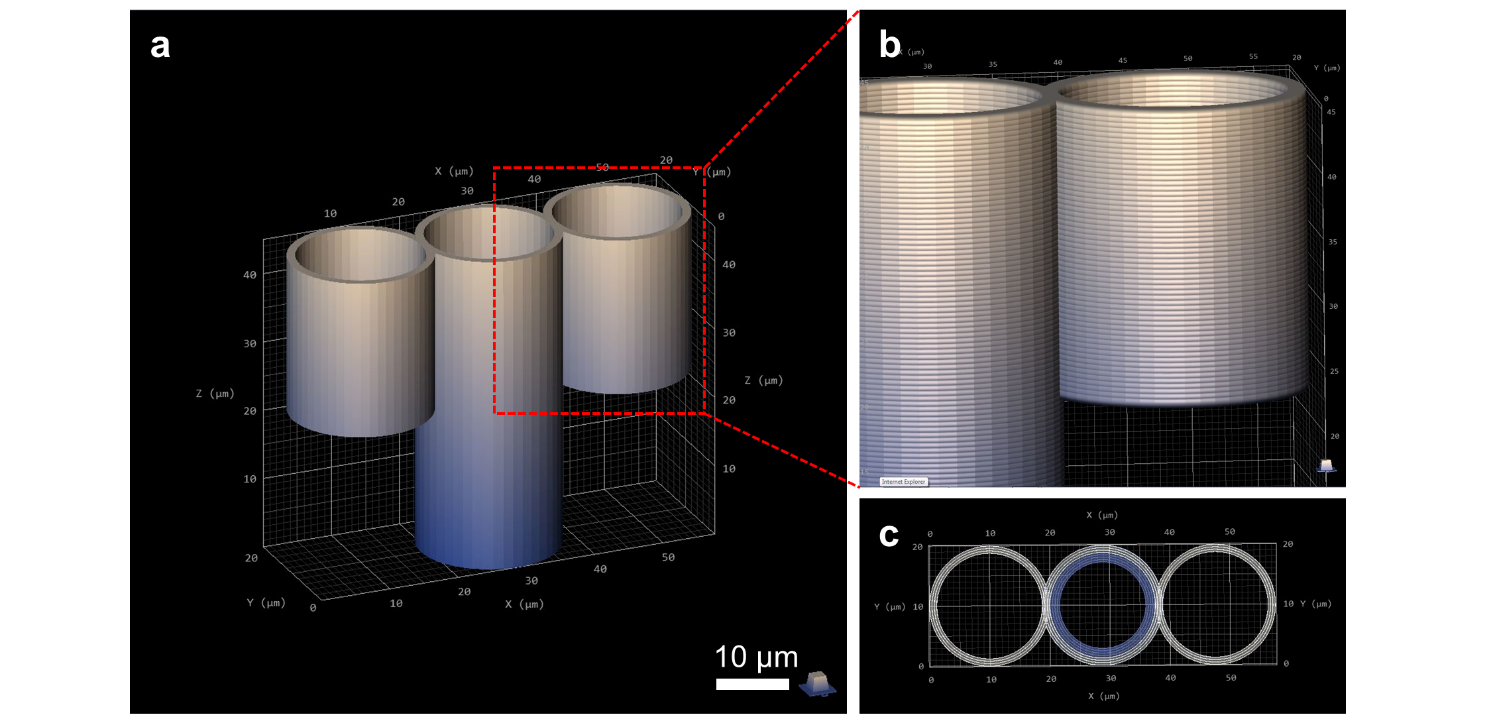


**Fig. S2**. Printing process of the micro-rocket. (a) Micro-rocket shown in DeScribe software. (b) Slicing details. Slicing distance: 400 nm. (c) Filling details for every slicing layer. Contour filling distance: 400 nm.


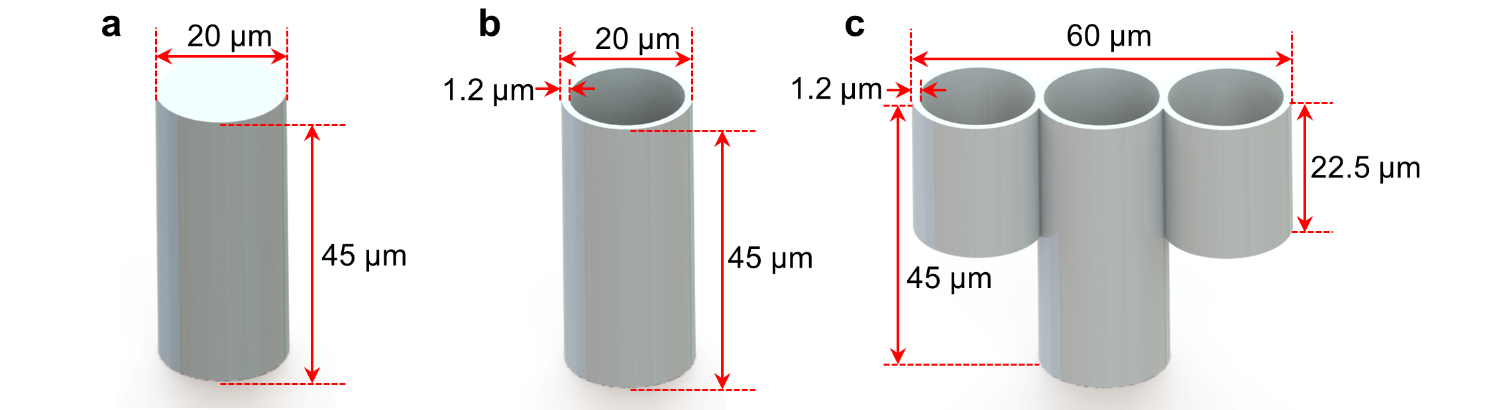


**Fig. S3**. 3D structure of designed micro-rod, micro-tube and micro-rocket. (a) Micro-rod with length of 45 µm and diameter of 20 µm. (b) Micro-tube with length of 45 µm, diameter of 20 µm and wall thickness of 1.2 µm. (c) Micro-rocket with length of 45 µm, width of 60 µm and height of 20 µm. A central 45-µm-high tube and two side 22.5-µm-high tubes, with 20-µm diameter and 1.2-µm-thick wall, make up the micro-rocket.


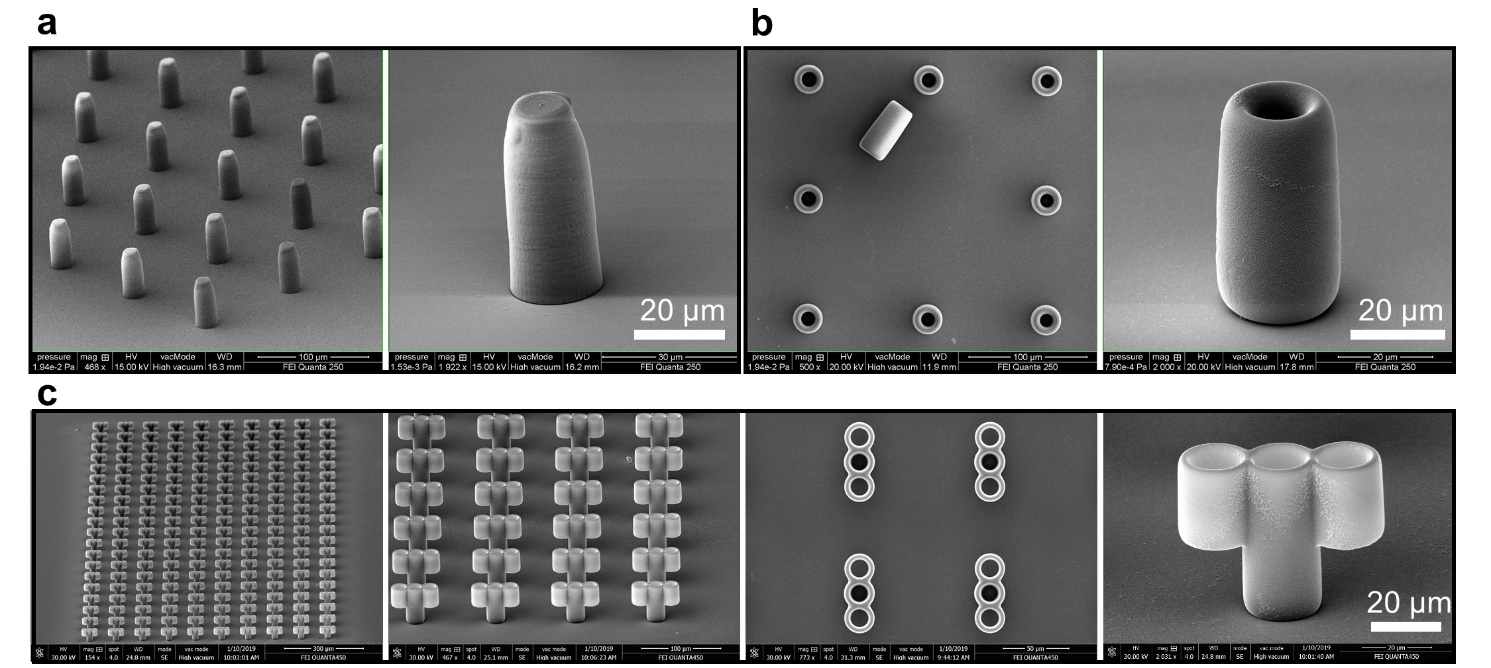


**Fig. S4**. SEM images of the micro-rod, micro-tube and micro-rocket. (a) An array of micro-rods and a single micro-rod. (b) An array of micro-tubes and a single micro-tube. (c) Top view and lateral view of the 200 micro-rockets array; A single micro-rocket with three nozzles.


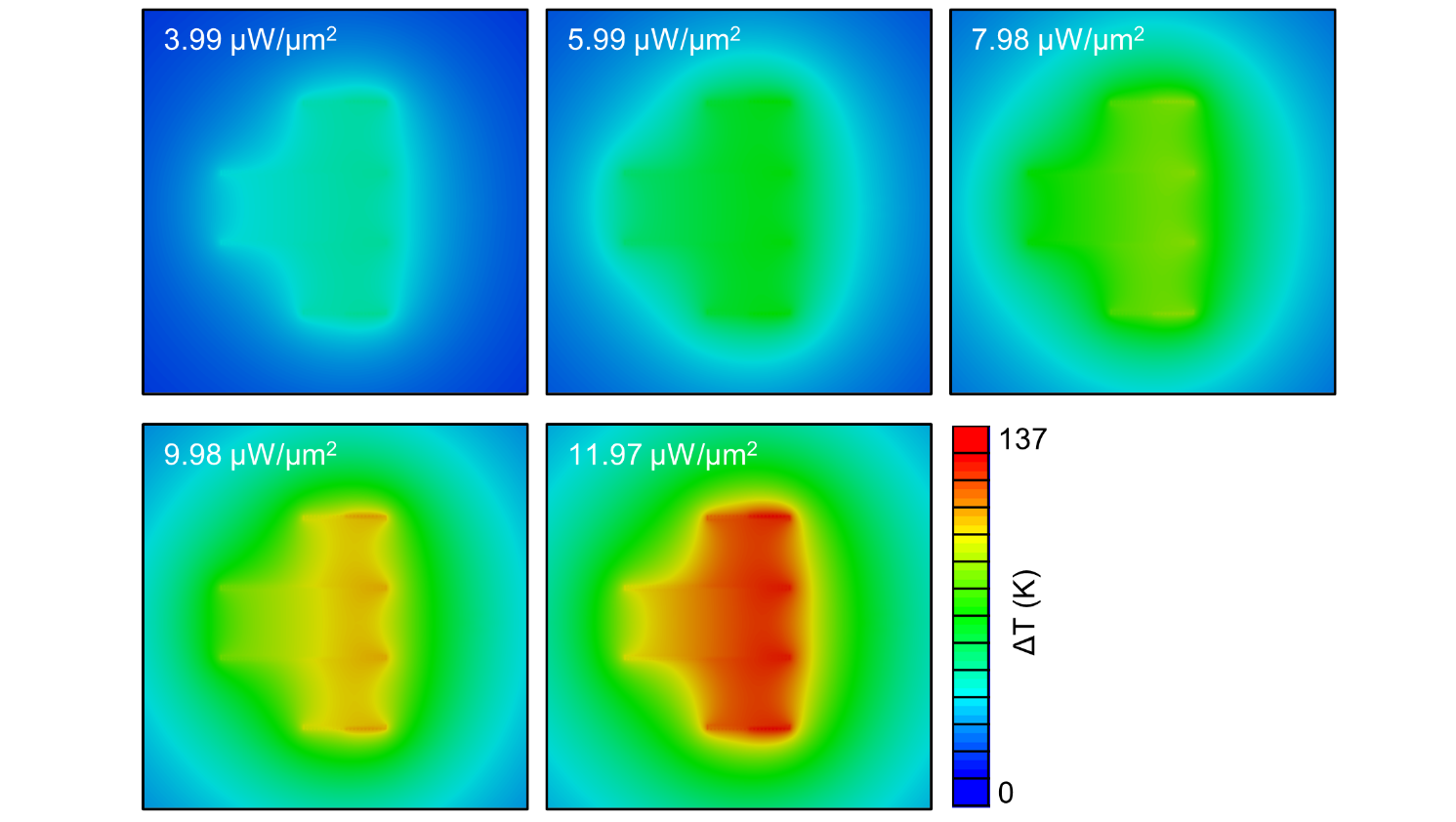


**Fig. S5**. Theoretically simulated temperature distribution on the micro-rocket under the irradiation of different laser power.


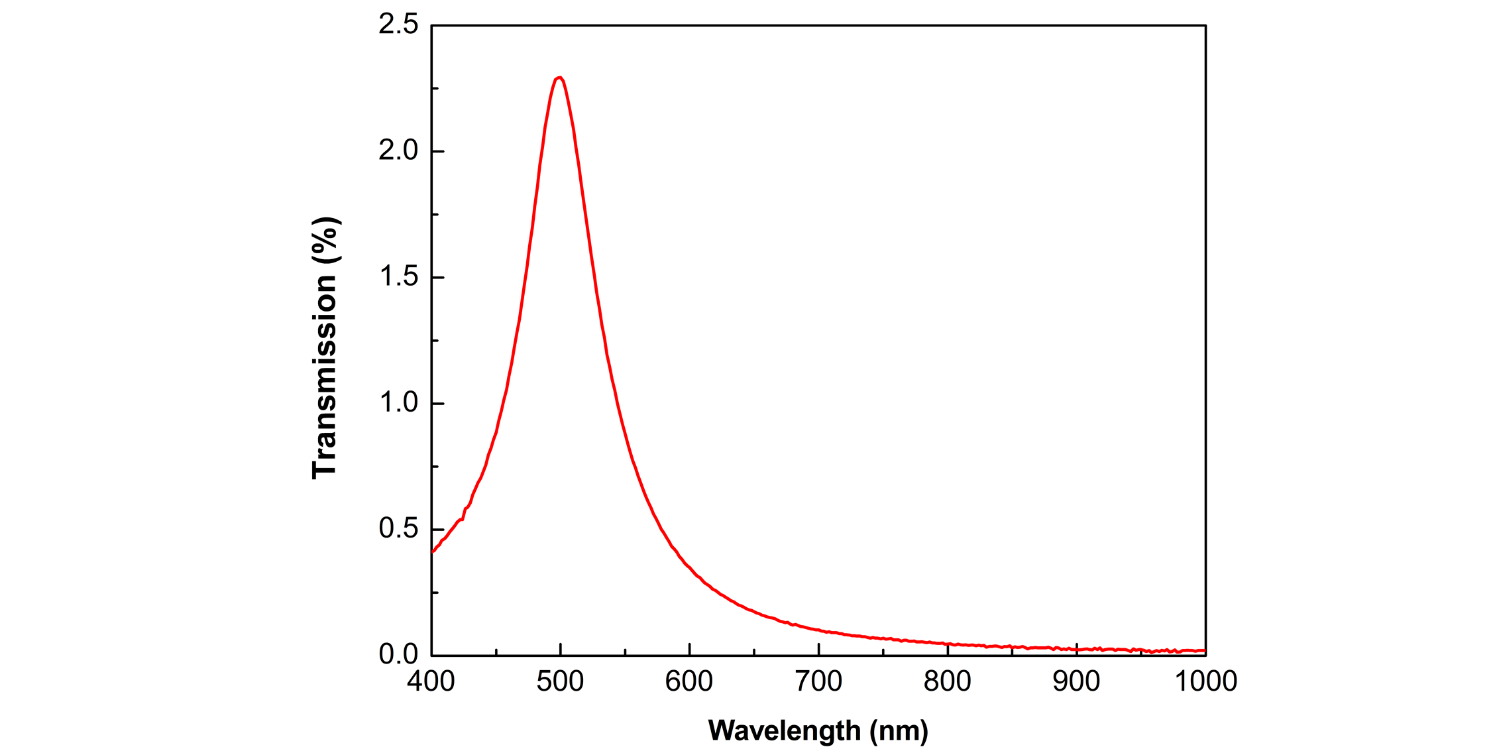


**Fig. S6**. Optical transmission for the 100 nm Au layer.


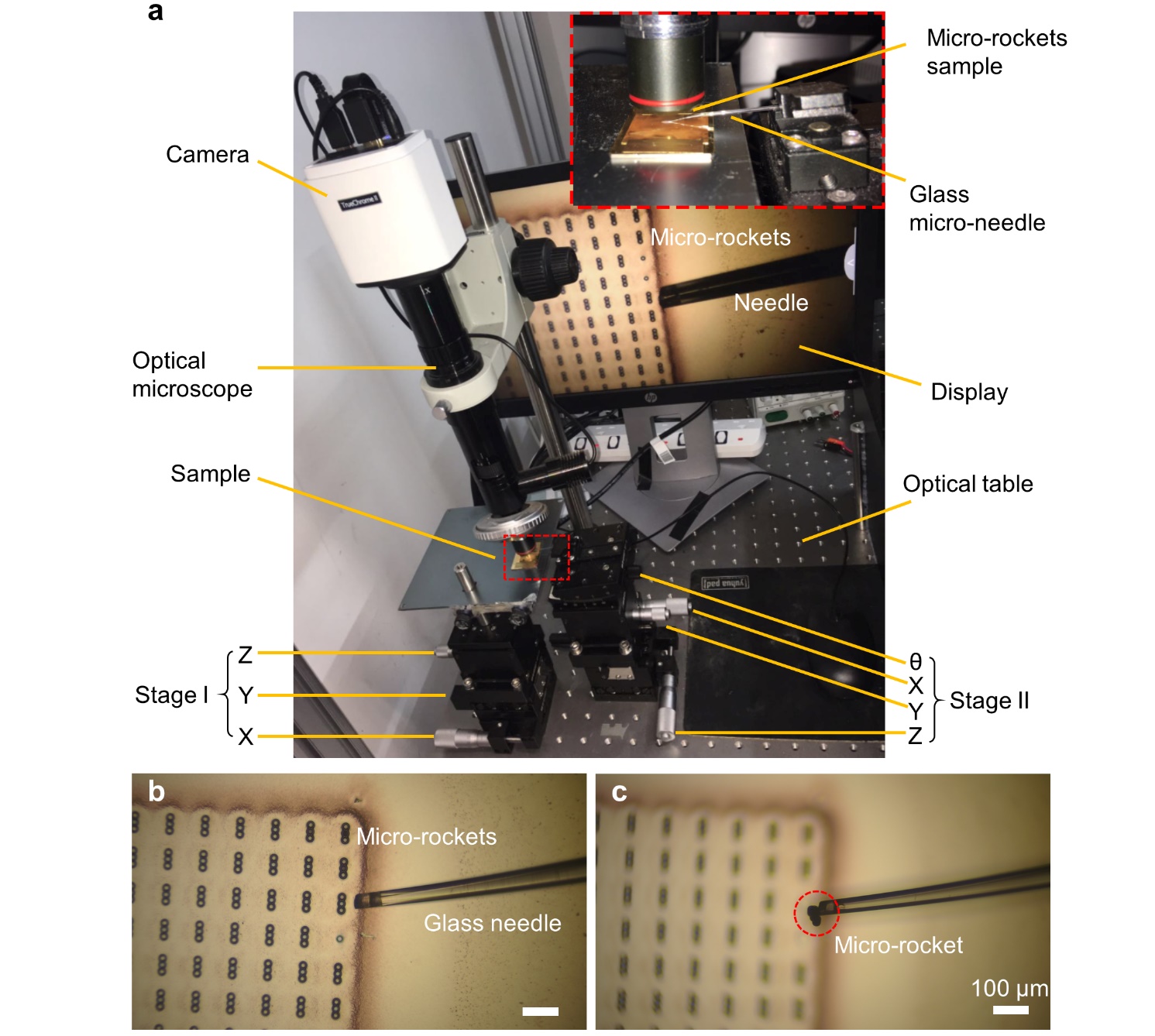


**Fig. S7**. Micro-manipulation system for micro-rockets’ transfer. (a) System image. (b) Pushing micro-rocket by a glass needle. (c) A rocket is attached on the needle.


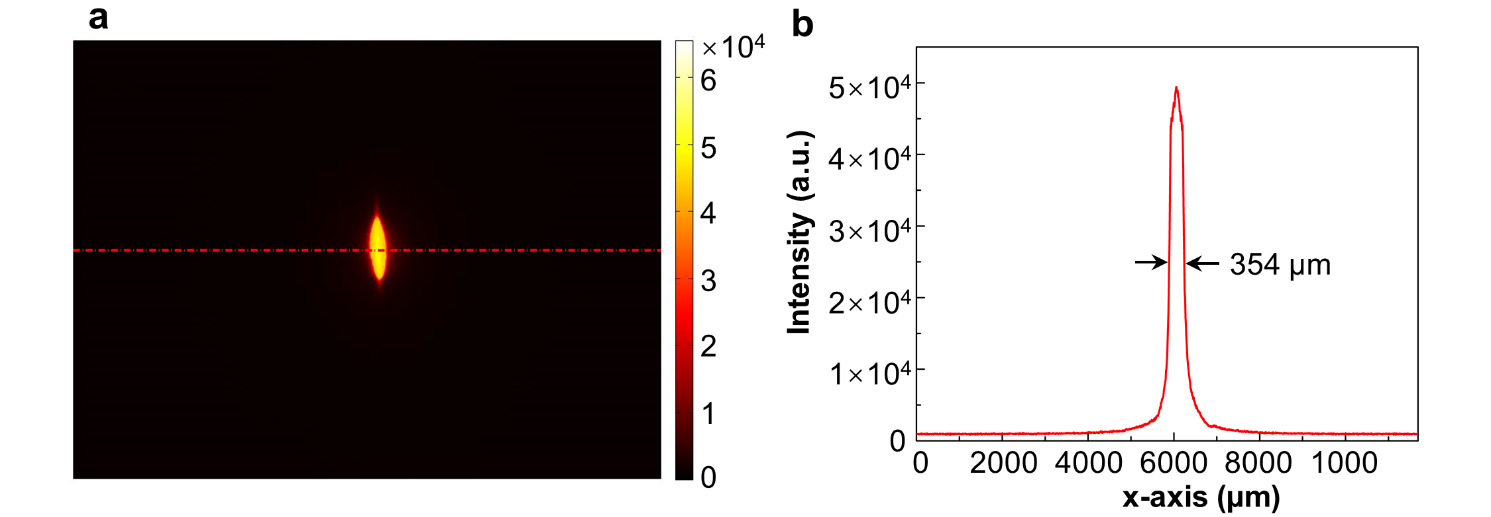


**Fig. S8**. Spot size of the excitation laser. (a) Image of the excitation laser beam. The 1-W laser with 808-nm wavelength irradiated on the screen and was observed by a CCD camera. (b) The intensity distribution along the central red dotted line in (a). Laser spot size can be confirmed by the full width at half maximum (FWHM). The FWHM is 354 µm at the distance of excitation. The spot area is approximately calculated according to the square area.

**References**

1. Tottori, S. *et al.* Magnetic Helical Micromachines : Fabrication , Controlled Swimming , and Cargo Transport. *Adv. Mater.* **24**, 811–816 (2012).

2. Gao, W. *et al.* Bioinspired helical microswimmers based on vascular plants. *Nano Lett.* **14**, 305–310 (2014).

3. Jurado-Sánchez, B., Wang, J. & Escarpa, A. Ultrafast Nanocrystals Decorated Micromotors for On-Site Dynamic Chemical Processes. *ACS Appl. Mater. Interfaces* **8**, 19618–19625 (2016).

4. Solovev, B. A. A., Sanchez, S., Pumera, M., Mei, Y. F. & Schmidt, O. G. Magnetic Control of Tubular Catalytic Microbots for the Transport , Assembly , and Delivery of Micro-objects. *Adv. Funct. Mater.* **20**, 2430–2435 (2010).

5. Kaynak, M. *et al.* Acoustic actuation of bioinspired microswimmers. *Lab Chip* **17**, 395–400 (2017).

6. Wang, W., Castro, L. A., Hoyos, M. & Mallouk, T. E. Autonomous motion of metallic microrods propelled by ultrasound. *ACS Nano* **6**, 6122–6132 (2012).

7. Wu, Z. *et al.* Superfast Near-Infrared Light-Driven Polymer Multilayer Rockets. *Small* **12**, 577–582 (2016).

8. Rao, Q., Si, T., Wu, Z., Xuan, M. & He, Q. A Light-Activated Explosive Micropropeller. *Sci. Rep.* **7**, 1–9 (2017).
